# Liver Resections for Metastases from Intraabdominal Leiomyosarcoma

**DOI:** 10.1155/1999/67324

**Published:** 1999-07

**Authors:** Antonio Nocchi Kalil, Bianca De Lourdes Pereira, Marcia Cristina Lima Brenner, Luiz Pereira-Lima

**Affiliations:** 1 Departament of Surgery of Fundação Faculdade Federal de Ciências Médicas de Porto Alegre (FFFCMPA) d'Université of Hepatobiliary Surgery University of Paris XI Brazil; 2 Medical student at FFFCMPA Brazil; 3 FFFCMPA Univesidade Federal do Rio Grande do Sul (UFRGS) Brazil; 4 Prça Mauricio Cardoso 170-402 CEP 90570-010 Rio Grande do Sul Porto Alegre Brazil

## Abstract

This paper discusses liver resection for intraabdominal leiomyosarcoma metastases as a therapy for carefully selected patients. Of the 83 hepatectomies performed from 1992 to 1996, five were resections for liver metastases due to intraabdominal leiomyosarcoma, in 3 patients. The surgical indication was single liver metastases, without any evidence of extrahepatic disease. No mortality occurred during surgery and the longest survival was 38 months. We concluded that liver resection for leiomyosarcoma metastases can be performed, allowing a long term survival in an occasional patient.


*HPB Surgery*, 1999, Vol. 11, pp. 261-264
Reprints available directly from the publisher
Photocopying permitted by license only

© 1999 OPA (Overseas Publishers Association) N.V.
Published by license under
the Harwood Academic Publishers imprint,
part of The Gordon and Breach Publishing Group.
Printed in Malaysia.

*Clinical Series*

### Liver Resections for Metastases from Intraabdominal Leiomyosarcoma

ANTONIO NOCCHI KALIL[a,*], BIANCA DE LOURDES PEREIRA[b], MARCIA CRISTINA LIMA BRENNER[b] and LUIZ PEREIRA-LIMA[c]

[a] *Associate Professor in the Departament of Surgery of Fundação Faculdade Federal de Ciências Médicas de Porto Alegre (FFFCMPA). Diplome d'Université of Hepatobiliary Surgery and Liver Transplants from the University of Paris XI;*
[b] *Medical student at FFFCMPA;* [c] *Full Professor at the FFFCMPA and Associate Professor at Universidade Federal do Rio Grande do Sul (UFRGS)*

**This paper discusses liver resection for intraabdominal leiomyosarcoma metastases as a therapy for carefully selected patients. Of the 83 hepatectomies performed from 1992 to 1996, five were resections for liver metastases due to intraabdominal leiomyosarcoma, in 3 patients. The surgical indication was single liver metastases, without any evidence of extrahepatic disease. No mortality occurred during surgery and the longest survival was 38 months. We concluded that liver resection for leiomyosarcoma metastases can be performed, allowing a long term survival in an occasional patient.**

*Keywords:* Leiomiosarcoma, liver metastases, liver resection, non-colorectal liver metastases

### INTRODUCTION

Leiomyosarcomas are malignant tumors of smooth muscles, occurring most commonly in the uterus, retroperitoneal region and extremities. These lesions correspond to 7% of the soft tissue tumors, most often affecting adult, female patients [1].

Metastatic dissemination occurs by haematogenous spread, mainly to the lungs and liver, and only occasionally to regional lymph nodes [2]. Liver metastases are usually observed in the reccurrence of visceral and retroperitoneal sarcomas they are often multiple and bilobar [3,4].

Some authors suggest that for certain non-colorectal tumors, including leiomyosarcoma, the resection of liver metastases might increase time of survival and/or promote substantial palliation [5-13].

This paper describes a group of patients with liver metastases due to leiomyosarcoma.

### PATIENTS AND METHODS

During the period from 1992 to 1997, 83 hepatectomies were performed by a single surgical team in the Department of Surgery, Fundação Faculdade Federal de Ciências Médicas de Porto Alegre (FFFCMPA). Five of them were liver resections

*Address for correspondence: Praça Mauricio Cardoso 170-402 CEP 90570-010, Porto Alegre, Rio Grande do Sul, Brazil.

for metastases due to leiomyosarcoma, performed in three patients. This group consisted of two men and one woman whose ages ranged from 41 to 56 (mean, 46.6), all of them caucasian.

The site of the primary tumor, the time of diagnosis of liver metastases and their location are summarized in Table I.

All the patients were submitted to a preoperative assessment which consisted of routine lab tests, liver function tests and imaging (ultrasonography-US and computed tomography-CT).

Liver resection was indicated for a single metastasis from leiomyosarcoma, without any evidence of disease at another site.

Hepatectomy was performed by a bilateral subcostal incision, with afferent vascular exclusion in four resections and selective clamping in one hepatectomy (patient 3, the first resection). Subhepatic silicone drains were used in all patients. One patient required chest drainage (patient 3).

Surgical mortality was defined by death up to the 30th day post-surgery. Time of survival was computed in months (from date of liver resection up to the latest visit). Reccurrence was defined by abnormalities in computed tomography, ultrasonography, biopsy or bone radionuclide scanning.

### RESULTS

The presence or absence of reccurrence after liver resection, the course and survival of patients are illustrated in Figure 1.

Two segmentectomies were performed in patient 1. Up to the present time, this patient,

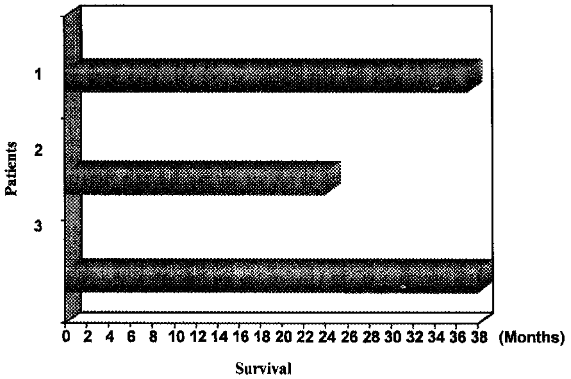

FIGURE 1 Post-resection survival and evolution of liver metastases due to leiomyosarcoma 1-Patient alive, without reccurrence at present, after re-hepatectomy at 25 months due to hepatic reccurrence; 2-Died. Extra-hepatic reccurrence at 4 months post-hepatectomy; 3-Died. Submited to re-hepatectomy at 12 months due to hepatic reccurrence and, at 24 months, presented new hepatic reccurrence.

is alive and asymptomatic, having adjuvant chemotherapy after the second resection, with a survival of 37 months.

Left lobectomy was performed in patient 2, who was submitted to radiation therapy and chemotherapy five months after liver resection, due to bone metastasis.

Bi-segmentectomy was performed in patient 3. He survived for 38 months with reccurrence in the liver after the second operation.

No mortality occurred in surgery, and the transfusion of blood products required ranged from 0 to 1000 ml (mean, 250 ml).

### DISCUSSION

An assessment of risk factors for the development of metastases due to soft tissue sarcomas

TABLE I Study of primary tumor and metastases

| Patients | Age (years) | Site of primary tumor | Type of metastasis | Location of metastases |
|---|---|---|---|---|
| 1 | 41 | S.B.* | 1) Synchronous<br>2) Metachronous | 1) Segment V<br>2) Segment VIII |
| 2 | 56 | Uterus | Metachronous | Segments II and III |
| 3 | 43 | Stomach | 1) Synchronous<br>2) Metachronous | 1) Segments V and VI<br>2) Segments VII and VIII |

*S.B. – Small bowel.

is dependent on 3 factors: histological grade, histological type and primary tumor site [3].

Leiomyosarcomas constitute 7% of the histological types, often affecting the uterus, stomach and small bowel. The most undifferentiated tumors, with the worst prognosis, are found in the retroperitoneal sites [1,3].

Recent studies indicate the liver as a preferential site for metastases from visceral and retroperitoneal leiomyosarcomas [3,4,14]. Most of these metastases are multiple and bilobar [12], and 50% of the patients who developed them did so in the first year after diagnosis. This is the period during which one must carefully assess the liver of patients with intra-abdominal leiomyosarcoma [3,4]. Once the diagnosis of liver metastases has been established, the type and histological grade, as well as disease-free time after resection of the primary tumor do not appear to influence patient survival [3].

Chemotherapy, systemic or intra-arterial, does not have a substantial impact on the treatment of liver metastases from soft tissue sarcomas [3,4,15]. However, Mavligit *et al.* [4] report their experience with 14 patients who had gastrointestinal leiomyosarcoma, treated by hepatic chemoembolization, using a mixture of polyvinyl alcohol with cisplatinum powder (150 mg), followed by intrahepatic arterial infusion of vinblastin (10 mg/m$^2$). In this series, all the patients had unresectable liver metastases; however, one patient had a dramatic regression of the tumor, which enabled its resection.

From data obtained in series in which the time of survival after liver resection due to metastases from leiomyosarcoma was established, a mean time of 33 months was obtained, one patient presented a survival longer than 5 years [2-16]. Thus, it seems clear that the leiomyosarcomas constitute an indication for liver resection in the case of non-colonic metastases.

Based on these findings, we operated on 3 patients with liver metastases from leiomyosarcoma, and obtained a survival ranging from 24 to 38 months. Two patients presented with reccurrence of the disease in the liver, and both of them were operated on again, corroborating the data from the literature which reports a high rate of reccurrence of this type of tumor in the liver [3,6,13]. The worst prognosis, was in the case of the patient with a metastasis of non-digestive origin, who presented with an extrahepatic reccurrence less than 6 months after hepatectomy.

Thus, we concluded that the resection of liver metastases due to leiomyosarcoma could be performed, allowing a long-term survival in an occasional patient.

### *Acknowledgments*

This study was performed in the Department of Surgery of Fundação Faculdade Federal de Ciências Médicas de Porto Alegre (FFFCMPA) and Irmandade Santa Casa de Misericórdia de Porto Alegre.

### *References*

[1] Rosenberg, A. E. (1994). Skeletal system and soft tissue tumors. In: *Pathologic Basis of Disease*, edited by Cotran, R. S., Kumar, V. and Robbins, S. L. 5th edn., pp. 1268-1269. Philadelphia: W.B. Saunders Co.
[2] Rodríguez López, I., Otero Echart, M., Carballo Fernández, C., Pérez Becerra, E., Casal Rubio, M. and Barrio Gómez, E. (1986). Leiomiosarcoma del intestino delgado con metástasis hepáticas y eosinofilia. *Revista Española de Enfermedades del Aparello Digestivo*, **70**(2), 165-68.
[3] Jaques, D. P., Coit, D. G., Casper, E. S. and Brennan, M. F. (1995). Hepatic metastases from soft-tissue sarcoma. *Annals of Surgery*, **221**(4), 392-97.
[4] Mavligit, G. M., Zukwisk, A. A., Ellis, L. M., Chuang, V. P. and Wallace, S. (1995). Gastrointestinal leiomyosarcoma metastatic to the liver-durable tumor regression by hepatic chemoembolization infusion with cisplatin and vinblastine. *Cancer*, **75**(8), 2083-2088.
[5] Coboum, C. S., Makowka, L., Langer, B., Taylor, B. R. and Falk, R. E. (1987). Examination of patient selection and outcome for hepatic resection for metastatic disease. *Surgical Gynecology and Obstetrics*, **165**, 239-46.
[6] González, E. M., Aguirre, J. I., Garcia, J. I. G., Vorwald, P., Martinez, J. B., Selas, P. R., Sanz, R. G., González, J. S., González-Pinto, I., Benito, F. E. and Segurola, C. L. (1992). Surgical treatment of hepatic metastases from malignant neoplasms of noncolorectal origin. *European Clinics I*, 71-75.
[7] Lee, Y. T. (1983). Regional management of liver metastases. *Cancer Investigation*, **1**(4), 321-332.
[8] Okuyama, K., Isono, K., Iee-Kung, J., Onoda, S., Ochiai, T., Yamamoto, Y., Koide, Y. and Satoh, H. (1985).

Evaluation of treatment of gastric cancer with liver metastasis. *Cancer*, **55**, 2498–2505.
[9] Pommier, R. F., Woltering, E. A., Campbell, J. R. and Fletcher, W. S. (1987). Hepatic resection for primary and secondary neoplasms of the liver. *American Journal of Surgery*, **153**, 428–433.
[10] Stehlin, J. S. Jr., De Ipolyi, P. D., Greeff, P. J., Mcgraff, C. J. Jr. Davis, B. R. and Mcnary, L. (1988). Treatment of cancer of the liver: twenty years' experience with infusion and resection in 414 patients. *Annals of Surgery*, **208**, 23–35.
[11] Wolf, R. F., Goodnight, J. E., Krag, D. E. and Schneider, P. D. (1991). Results of resection and propoused guidelines for patient selection in instances of non-colorectal hepatic metastases. *Surgical Gynecology and Obstetrics*, **173**, 454–460.
[12] Attiyeh, F. F. and Wichern, W. A. Jr. (1988). Hepatic resection for primary and metastatic tumors. *American Journal of Surgery*, **156**, 368–373.
[13] Bines, S. D., England, G., Deziel, D. J., Witt, T. R., Doolas, A. and Roseman, D. L. (1993). Synchronous, metachronous and multiple hepatic resections of liver tumors originating from primary gastric tumors. *Surgery*, **114**, 799–805.
[14] Schwartz, S. I. (1995). Hepatic resection for noncolorectal nonneuroendocrine metastases. *World Journal of Surgery*, **19**, 72–75.
[15] Sesto, M. E., Vogt, D. P. and Hermann, R. E. (1987). Hepatic resection in 128 patients: a 24-year experience. *Surgery*, 5(102), 846–851.
[16] Lacaine, F., Flamant, Y., Boudet, M. J. and Hay, J. M. (1994). Surgical treatment of noncolorectal hepatic metastases: long-term results following 91 liver resections. *French Association of Surgical Research. World Congress of the International Hepato-Pancreato-Billiary Association*, Abstract Book, pp. 16–17.

**Invited commentary to**: Kalil AN, de Lourdes Pereira B, Brenner MCL, Pereira-Lima L. Liver resections for metastases from intraabdominal leiomyosarcoma. HPB Surgery

### INVITED COMMENTARY

The incidence of isolated hepatic metastases from cancers of the colon or rectum is frequent enough to justify hepatic resection in selected patients. For other malignancies metastases to the liver only is quite a rare phenomen, which explains why the literature contains relatively few and almost anecdotal reports of long-term survival following hepatic resection of metastases from non-colorectal malignancies, such as intra-abdominal leiomycoscarcoma.

The present study has shown that long-term survival (at least for 3 years) is possible following resection of metastatic intra-abdominal leiomyosarcoma. A few other authors have reported similar findings with an occasional 5-year survivor, although none of 10 patients with liver metastases from visceral leiomyosarcoma survived for 5 years in the largest single series [1]. It should be emphasized that spread to the liver is the main factor determining survival for visceral sarcomas but not for sarcomas originating at other locations, with the exception of retroperitoneal sarcomas that spread as often to the liver as to the lungs [1]. The pattern of spread, and the likelihood of having metastases only in the liver, is of course influenced by the portal venous drainage of gastrointestinal organs.

Definite indications do not exist, but it would appear that a patient with isolated and resectable hepatic metastases from gastrointestinal leiomyosarcoma should undergo resection [2]. A prerequisite is that the resection can be undertaken with low morbidity and low mortality (well under 5%). Considering the relative inefficiency of chemotherapy for leiomyosarcoma, it is also important to be able to ensure clear resection margins. The difficulty to be confident about indications and prognostic factors is true for hepatic resection of all other non-colorectal secondaries and should, hopefully, lead to an international registry for patients undergoing liver resection for non-colorectal secondaries.

### *References*

[1] Jaques, D. P., Coit, D. G., Casper, E. S. and Brennan, M. F. (1995). Hepatic metastases from soft-tissue sarcoma. *Ann. Surg.*, **221**, 392–397.
[2] Wolf, R. F., Goodnight, J. E., Krag, D. E. and Schneider, P. D. (1991). Results of resection and proposed guidelines for patient selection in instances of noncolorectal hepatic metastases. *Surg. Gynecol. and Obstet.*, **173**, 454–460.

Karl-G. Tranberg
Department of Surgery
Lund Univeristy
Lund
Sweden

